# *De novo* Transcriptome Analysis of *Portunus trituberculatus* Ovary and Testis by RNA-Seq: Identification of Genes Involved in Gonadal Development

**DOI:** 10.1371/journal.pone.0128659

**Published:** 2015-06-04

**Authors:** Xian-liang Meng, Ping Liu, Fu-long Jia, Jian Li, Bao-Quan Gao

**Affiliations:** Key Laboratory of Sustainable Development of Marine Fisheries, Yellow Sea Fisheries Research Institute, Chinese Academy of Fishery Sciences, Qingdao, Shandong, People’s Republic of China; Zhejiang University, CHINA

## Abstract

The swimming crab *Portunus trituberculatus* is a commercially important crab species in East Asia countries. Gonadal development is a physiological process of great significance to the reproduction as well as commercial seed production for *P*. *trituberculatus*. However, little is currently known about the molecular mechanisms governing the developmental processes of gonads in this species. To open avenues of molecular research on *P*. *trituberculatus* gonadal development, Illumina paired-end sequencing technology was employed to develop deep-coverage transcriptome sequencing data for its gonads. Illumina sequencing generated 58,429,148 and 70,474,978 high-quality reads from the ovary and testis cDNA library, respectively. All these reads were assembled into 54,960 unigenes with an average sequence length of 879 bp, of which 12,340 unigenes (22.45% of the total) matched sequences in GenBank non-redundant database. Based on our transcriptome analysis as well as published literature, a number of candidate genes potentially involved in the regulation of gonadal development of *P*. *trituberculatus* were identified, such as *FAOMeT*, *mPRγ*, *PGMRC1*, *PGDS*, *PGER4*, *3β-HSD* and *17β-HSDs*. Differential expression analysis generated 5,919 differentially expressed genes between ovary and testis, among which many genes related to gametogenesis and several genes previously reported to be critical in differentiation and development of gonads were found, including *Foxl2*, *Wnt4*, *Fst*, *Fem-1* and *Sox9*. Furthermore, 28,534 SSRs and 111,646 high-quality SNPs were identified in this transcriptome dataset. This work represents the first transcriptome analysis of *P*. *trituberculatus* gonads using the next generation sequencing technology and provides a valuable dataset for understanding molecular mechanisms controlling development of gonads and facilitating future investigation of reproductive biology in this species. The molecular markers obtained in this study will provide a fundamental basis for population genetics and functional genomics in *P*. *trituberculatus* and other closely related species.

## Introduction

The swimming crab *Portunus trituberculatus* (Crustacea: Decapoda: Brachyura) is a commercially important crab species widely distributed in the estuary and coastal areas of Korea, Japan, China, and Southeast Asia [1]. This species is predominant in portunid crabs fisheries around the world and supports a large aquaculture industry in China. In 2010, *P*. *trituberculatus* production in China reached up to 91,050 tons and valued more than AUS$ 2.5 billion [2]. Owing to rapid development of the swimming crab culture industry, the demand for high-quality seeds has exceeded the supply. To improve the means of artificial seed production, it is very important to understand the regulatory mechanisms underlying reproductive development in this species.

The regulatory mechanisms implicated in crustacean reproductive development have long been of interest to biologists and aquaculture industry. Over the past few decades, extensive studies have been carried out and a variety of regulatory factors, such as methyl farnesoate, ecdysteroids, crustacean hyperglycemic hormones, biogenic amines and vertebrate-type steroids, have been identified and investigated in numerous crustacean species [3–13]. These studies have revealed a good picture of endocrine regulation for reproductive processes in crustacean species, however, the molecular mechanisms controlling gonadal development still remains poorly understood. The major obstacle in defining the molecular mechanisms is the lack of genetic and genomic information available for crustacean species. Transcriptome sequencing can yield a subset of genes from the genome that are functionally active in selected tissues [14], and is an effective way to discover genes participating in specific biological processes when genome sequence is not available [15,16]. Recently, the advent of massively parallel DNA sequencing technology (RNA-Seq), including ABI SOLiD, Roche 454 and Illumina Solexa platforms, have opened up the opportunities for exploring the transcriptome of non-model species at unprecedented sensitivity and depth. Using RNA-seq technology, transcriptome sequencing of reproductive tissues has been performed in several commercial crustacean species during the past three years [17–24], and many reproduction-related genes and pathways have been identified. However, no data is currently available on the gonad transcriptome of *P*. *trituberculatus*.

In the present study, we employed Illumina sequencing technology and *de novo* assembly to obtain the transcriptome of ovary and testis tissues in *P*. *trituberculatus* and discover genes potentially involved in ovarian and testicular development and maturation. To our knowledge, this work is the first report for transcriptome profile analysis of gonads in *P*. *trituberculatus*. This transcriptome dataset will provide valuable resources for unraveling the molecular mechanisms governing reproductive development in this species, and reference information for closely related crustacean species. Furthermore, a large number of markers potentially useful for the investigation of population genetics and molecular breeding strategies, including simple sequence repeats (SSRs) and single nucleotide polymorphisms (SNPs), were also reported.

## Materials and Methods

### Ethics statement

All the experimental procedures involving the handling and treatment of the crabs used in this study were approved by the Yellow Sea Fisheries Research Institute's Animal Care and Use Committee prior to initiation of experiments.

### Tissue collection

The female and male crabs used in this experiment were obtained from Haifeng Company, Weifang, China. Five crabs for each sex at 3- to 12-month age were collected every month, which covered individuals at different gonadal developmental stages from immature to spent. After transferred to the laboratory, the crabs were reared at the temperature of 23~26°C and the salinity of 30~32 for ten days. Then the crabs were placed in an ice bath until anesthetized, and about 50 mg (wet weight) of ovary or testis were dissected, snap-frozen in liquid nitrogen and stored at -80°C. The rest portion were fixed in Bouin’s solution for histological examination. Based on the external features (size, morphology and color) and histological configuration, ovarian development was classified into six stages [25]: Stage I (ovary is ribbonlike and transparent, main cell types are oogonia and pre-vitellogenic oocyte), Stage II (ovary is milk white, main cell types are endogenous vitellogenic and pre-vitellogenic oocyte), Stage III (ovary is buff and orange, main cell type is exogenous vitellogenic oocyte), Stage IV (ovary is deep orange, main cell types are exogenous vitellogenic and nearly mature oocyte), Stage V (ovary is deep orange, main cell type is mature oocyte), Stage VI (ovary is spent). Testicular development was classified into five stages [26]: Stage I (main cell type is spermatogonium), Stage II (main cell type is spermatocyte), Stage III (main cell type is spermatid), Stage IV (main cell type is sperm), Stage V (sperms have been expelled out). According to the staging results, ovary or testis tissues from three individuals at each developmental stages were subjected to RNA extraction.

### RNA extraction, cDNA library construction and sequencing

In order to obtain as many genes related to gonadal development as possible, samples of all the ovarian and testicular stages were used for RNA extraction. Total RNA was isolated from each sample using Trizol reagent (Invitrogen, Carlsbad, USA) and treated with RNase free DNase I (Promega, Madison, USA) following manufacturer’s protocol. RNA degradation and contamination were assessed using agarose gels (1%). Quantity and integrity of the RNA samples were determined using Nano Photometer spectrophotometer (Implen, Westlake Village, USA) and Bioanalyzer 2100 system (Agilent Technologies, Santa Clara, USA).

A total of 3 μg RNA was used for ovary or testis cDNA library construction. For ovary, 166.7 ng RNA from each crab at six ovarian stages (3 individuals per stage) were pooled together, and for testis, 200.0 ng RNA from each crab at five testicular stages (3 individuals per stage) were used to make a pool. The transcriptome libraries were generated with TruSeq RNA Sample Preparation Kit (Illumina, San Deigo, USA) according to the manufacturer’s instructions and two index codes were added in order to attribute sequences to ovary or testis samples. Then the clustering of the index-coded samples was conducted with TruSeq PE Cluster Kit (Illumina, San Francisco, USA) on a cBot Cluster Generation System following the manufacturer’s recommendations. After cluster generation, the ovary and testis libraries were sequenced on Illumina Hiseq 2000 platform using paired-end technology.

### Data processing, assembly and functional annotation

Raw image data file from Illumina HiSeq 2000 was transformed to raw reads by CASAVA base recognition and stored in fq format files. To obtain high-quality clean data, in-house perl scripts were used to filter the raw reads which trimmed the adapter sequences, removed the reads containing poly-N and the reads with low quality (quality value of over 50% bases of the read was less than 5).

*De novo* transcriptome assembly was accomplished with Trinity software [27], by which transcripts and unigenes (the longest transcript of a set of transcripts that appear to stem from the same transcription locus) were obtained. Gene functions of all the assembled unigenes were annotated based on the following databases with a cut-off E value of 1.0×10^–5^: Nr (NCBI non-redundant protein sequences); Pfam (Protein family); Swiss-Prot (A manually annotated and reviewed protein sequence database). Blast2go (http://www.BLAST2go.org/) and WEGO software (http://wego.genomics.org.cn/cgi-bin/wego/index.pl) were used to get the Gene ontology (GO) (http://www.geneontology.org/) annotation and GO functional classification for the unigenes. Mapping of the unigenes to KEGG (Kyoto Encyclopedia of Genes and Genomes) pathways were performed with KEGG Automatic Annotation Server (KAAS) (http://www.genome.jp/kegg/kaas/) [28].

### Sequence mapping and differential expression analysis

The assembled transcriptome was used as reference database, and gene expression levels were determined for each sample. Briefly, clean reads were mapped back to the reference transcriptome by Bowtie v0.12.9 and read count for each gene was obtained from the mapping results by RSEM [29]. And then the data was normalized for variation in sequencing depth with RPKM (Reads Per Kilobase of exon model per Million mapped reads) method [30] and input into DEGseq (2010) R package [31] for differential expression analysis. *P* value was adjusted using *q* value [32]. Q value<0.005 and |log2 (fold change) |>1was defined as the threshold for significant differential expression.

### Molecular markers detection

The MIcroSAtellite (MISA, http://pgrc.ipk-gatersleben.de/misa/misa.html) tool was used to identify the SSR markers in the unigenes. The minimum number of repeat units for di-, tri-, tetra-, penta- and hexa-nucelotide motifs were set as 6, 5, 5, 5 and 5, respectively. For putative SNP identification, clean reads were aligned to the reference transcriptome with SOAP2 software [33]. Based on the alignment results, SOAPsnp package [34] was employed to call SNPs. The SOAPsnp results were filtered using the following standards: base quality score is not less than 20 and distance between two SNPs is greater than 5.

### Quantitative real-time PCR confirmation of Illumina sequencing data

In order to validate the Illumina sequencing data, twelve differentially expressed genes between ovary and testis were chosen for quantitative real-time PCR analysis with the same RNA samples for transcriptome analysis. The PCR reactions were run in ABI 7500 real-time PCR system (Applied Biosystems, Foster City, USA) using QuantiFast SYBR Green PCR Kit (Qiagen, Hilden, Germany) in 25 μl reaction mixture with 20 ng cDNA as template. The *β-actin* was used as the reference gene to normalize expression levels of the tested genes [35], and relative gene expression was analyzed using the 2^-△△CT^ method [36]. All the primers used were manufactured by Invitrogen (Shanghai, China) (S2 Table). All measurements were performed in triplicates.

### SNP validation

To verify the predicted SNPs identified in the assembled transcripts, 12 transcripts containing 35 potential SNPs were selected for validation using the same cDNA samples as for transcriptome analysis. Primers were designed within the flanking regions of the SNPs using primer 3 [37] and were listed in S3 Table. After examining the specificity and molecular weight with agarose gel electrophoresis, PCR products were directly sequenced using both forward and reverse primers at Invitrogen Company (Shanghai, China). Sequencing chromatograms were analyzed using BioEdit software (http://www.mbio.ncsu.edu/bioedit/bioedit.html).

## Results and Discussion

### Illumina sequencing and *de novo* assembly

In order to obtain an overview of gonad transcriptome of *P*. *trituberculatus* and identify genes involved in gonadal development, two cDNA libraries were prepared from pooled RNA extracts of ovary and testis at different development stages and sequenced using the Illumina Solexa platform. The transcriptome sequencing generated 135,337,108 raw reads in total (61,114,664 and 74,222,444 reads from ovary and testis, respectively). After trimming adapters and removing low-quality reads, the two sequence datasets were reduced to 5.84 and 7.04 GB for ovary and testis, respectively. Detailed results of the sequencing and assembly are shown in Table 1. All the reads were deposited in the Short Read Archive (SRA) of the National Center for Biotechnology Information (NCBI) with the accession number SRR1920180 (testis) and SRR1920182 (ovary).

**Table 1:** Summary statistics of *P*. *trituberculatus* gonad transcriptome sequencing and assembly.

**Raw results**	
Number of ovary raw reads	61,114,664
Number of testis raw reads	74,222,444
Numer of total raw reads	135,337,108
Number of ovary clean reads	58,429,148
Number of testis clean reads	70,474,978
Numer of total clean reads	128,904,126
**Assembly results**	
Number of transcripts	80,527
Average length of transcripts (bp)	1,053
Minimum transcripts (bp)	201
Maximum transcripts (bp)	36,343
N50	2,439

The *de novo* transcriptome assembly performed with Trinity using both ovary and testis reads (128,904,126 reads in all) generated a total of 80,527 transcripts. The length distribution of the assembled transcripts is as shown in S1 Fig. The average length of the transcripts obtained, ranging from 201 to 36,343 bp, was 1,053 bp. The assembly program produced a substantial number of long sequences, i.e. 21,134 transcripts were longer than 1,000 bp, accounting for 26.24% of total transcripts, and 11,468 transcripts (14.24%) were longer than 2,000 bp. Long sequences with high quality enable us to gain more information on genes. Therefore this transcriptome dataset will provide a valuable resource for future analysis of genes associated with reproduction and other economic traits.

### Annotation and functional classification

**Fig 1:**
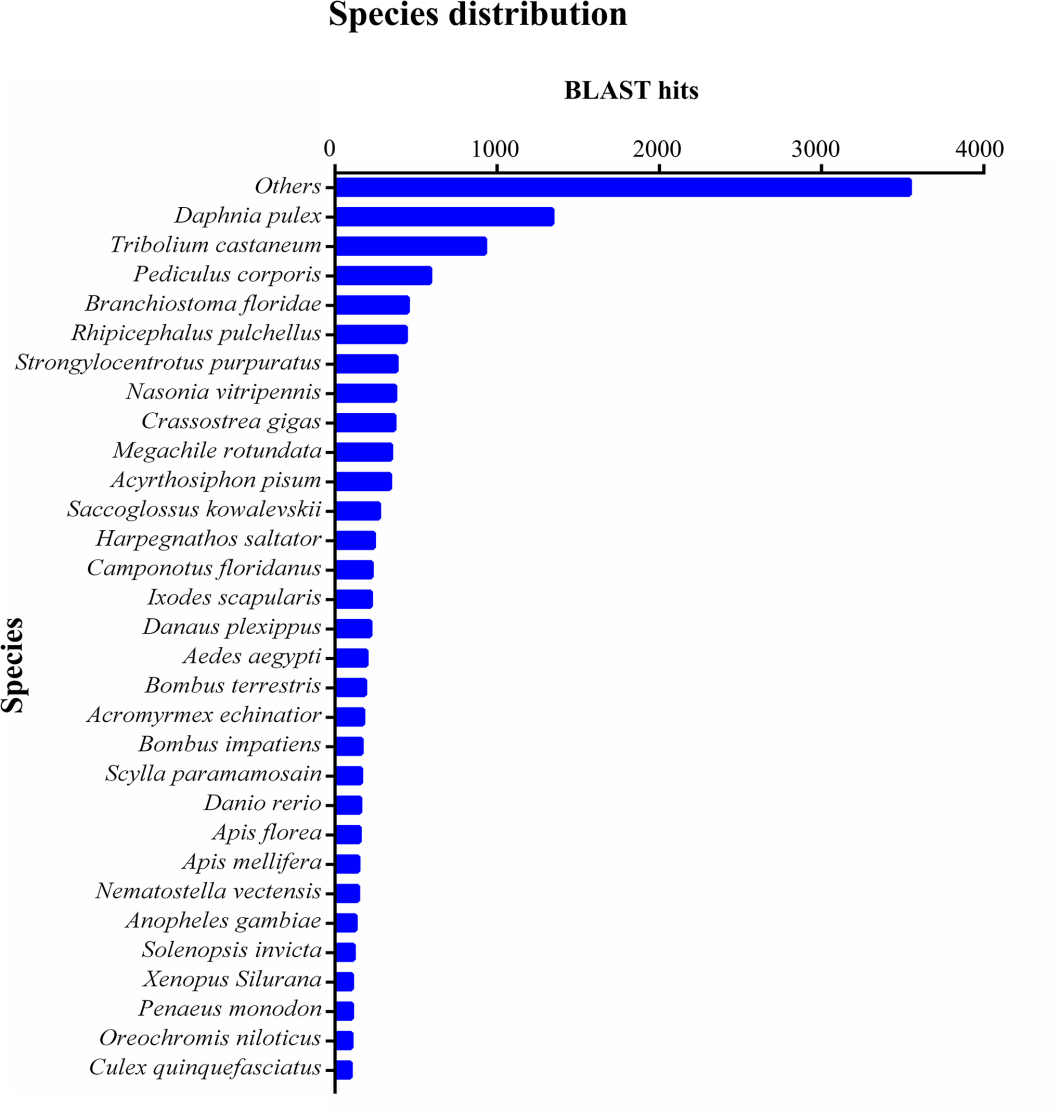
Species distribution of the BLASTx matches of the gonad transcriptome unigenes. Each bar of the histogram indicates the number of top-BLAST matches (the matches with the lowest E-value for each unigene) against the Genbank non-redundant (Nr) protein database to various species.

After eliminating low-quality and short-length sequences, 54,960 unigenes were subjected to annotation analysis by matching sequences against Nr, Pfam and Swiss-prot databases. 12,340 unigenes (22.45% of the total) can be matched in Nr database, 14,770 unigenes (26.87% of the total) matched Pfam, and 10,236 unigenes (18.62% of the total) matched in Swiss-prot. These annotated unigenes made a substantial contribution to *P*. *trituberculatus* sequence database and established the basis for future investigations on specific molecular processes and functions in this species. It was noted that a large proportion of the unigenes (77.55%) did not give any BLASTx hit, which could be partly due to the overall short length of these unigenes or due to the limited genomic information available for decapod crustaceans. The high proportion of unannotated sequences was also observed in previous transcriptome analysis of other crustaceans [19,22].The BLASTx top-hit species distribution of the 12,340 annotated unigenes showed highest homology to the microcrustacean *Daphnia pulex*, followed by *Tribolium castaneum*, *Pediculosis corporis*, *Branchiostoma floridae*, *Rhipicephalus pulchellus* and *Strongylocentrotus purpuratus* (Fig 1). Not surprisingly, the largest number of the unigenes was matched with the model species *D*. *pulex*, since it was the only crustacean species whose whole-genome sequencing had been completed [38].

**Fig 2:**
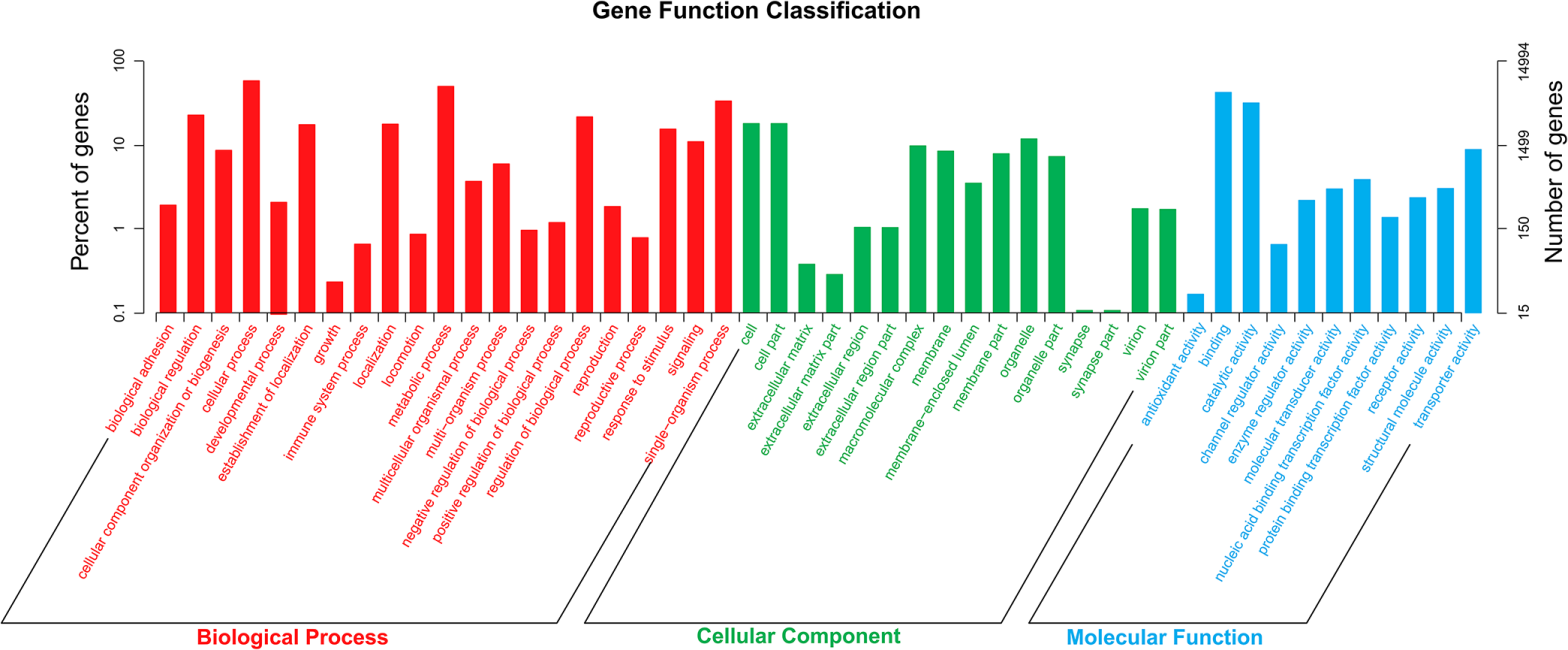
Gene ontology (GO) assignment of assembled unigenes of *P*. *trituberculatus*. GO terms were processed by Blast2Go and categorized at 2^nd^ level under three main categories (biological process, cellular component, and molecular function).

Gene ontology (GO) assignment programs were utilized for functional categorization of the assembled unigenes. A total of 14,994 unigenes were grouped into 55 subcategories under three main ontologies (molecular functions, cellular components and biological processes) by BLAST2GO suite (Fig 2). Of these unigenes, 12,514 (83.46%) were assigned to molecular function, followed by 11,234 (74.92%) to biological processes and 9,388 (62.61%) to cellular components. Within the molecular function, binding (52.82%) and catalytic activity (39.30%) constituted the majority of the category. In cellular components category, cell (49.79%), cell part (49.76%) and organelle (33.25%) comprised the largest proportion. Under biological processes category, the predominant GO terms were grouped in cellular process (52.82%) and metabolic process (54.69%). This GO assignment result was similar to the previously sequenced *Eriocheir sinensis* testis transcriptome in which cell, cell part, binding, catalytic activity cellular process and metabolic process represented the most abundant classifications [22].

**Fig 3:**
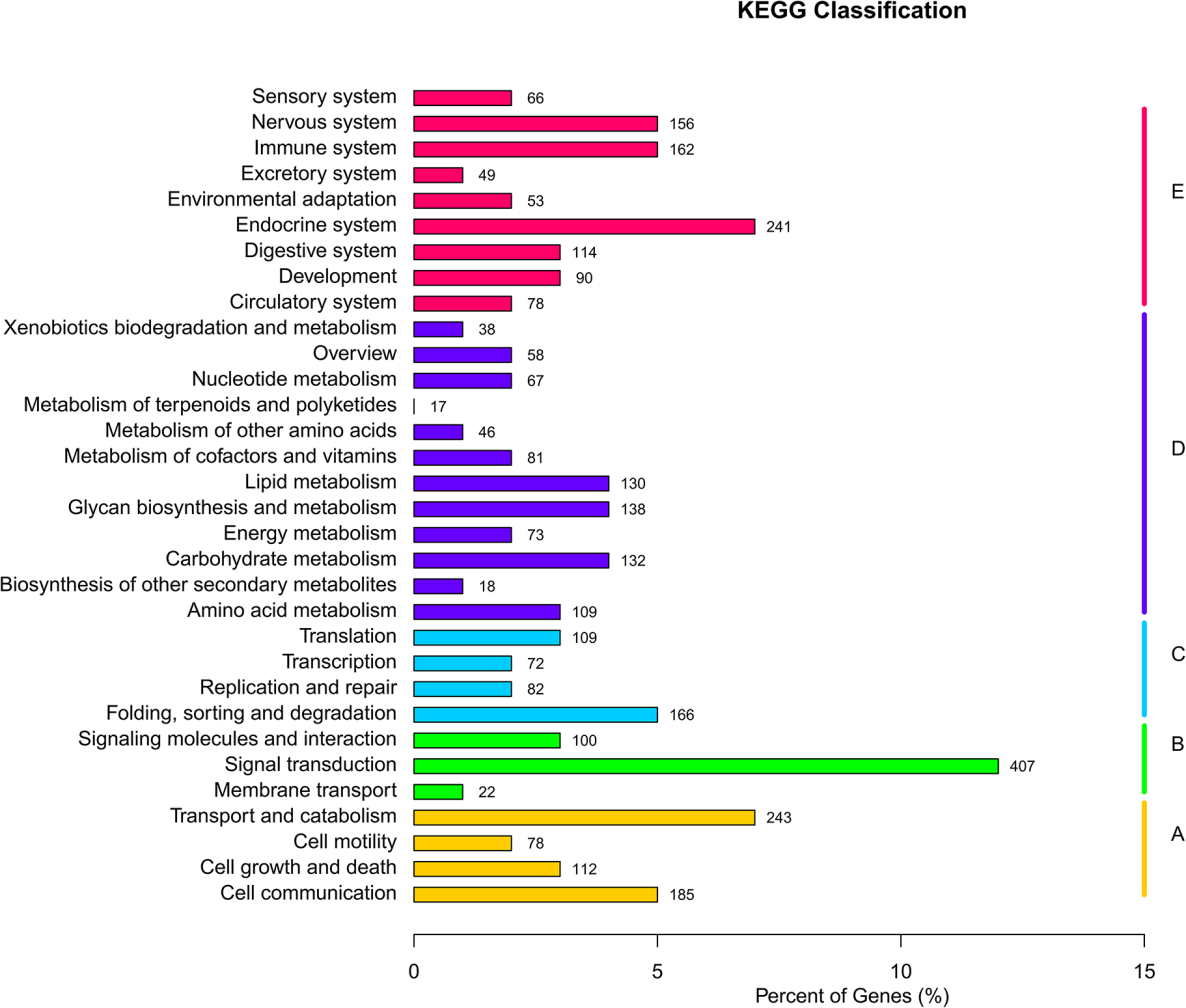
KEGG classification of the unigenes. 3,424 unigenes were assigned to 32 KEGG categories.

To identify the biological pathways active in *P*. *trituberculatus* gonads, all unigenes were mapped to the reference canonical pathways in KEGG database. This database contains functional information on metabolic pathways or regulatory networks of genes and interacting molecules in cells, which helps to study the complex biological behaviors of genes. Totally, 3,424 unigenes were mapped to 244 KEGG pathways within 32 categories, and among these pathways, several signaling pathways well-documented to be essential in gonadal development and maturation were found, including progesterone-mediated oocyte maturation pathway, GnRH signaling pathway, insulin signaling pathway, transforming growth factor β (TGF-β) pathway, the wingless-type MMTV integration site family (Wnt) pathway and phosphatidylinositol 3 kinase (PI3K)/Akt pathway [23,39–41]. These pathways were assigned to the KEGG categories of “signal transduction” and “endocrine system”, both of which were among the most represented categories (Fig 3), indicating the significance of signal transduction systems and endocrine regulation in gonad development and function in *P*. *trituberculatus*. The GO and KEGG annotations were helpful for identifying potential genes with specific function from a large-scale transcriptome database, and meanwhile provided a substantial resource for studying significant processes, functions and pathways during gonadal development in *P*. *trituberculatus*.

### Candidate genes involved in the regulation of gonadal development

The developmental processes of gonad in crustaceans is exquisitely orchestrated by a variety of regulatory factors, such as hormones and neurotransmitters [42–45]. Although the effects of these factors on gonadal development have been extensively investigated [46–49], the molecular mechanisms controlling the biosynthesis of the factors and mediating their physiological functions, are still largely unknown. In this gonad transcriptome of *P*. *trituberculatus*, we found a number of genes participating in the synthesis and metabolism of hormones, and genes encoding the receptors for hormones and biogenic amines important for gonadal development and maturation (Table 2). The identification and characterization of these genes will facilitate researches on reproductive endocrinology at the molecular level in this species.

**Table 2:** Candidate genes involved in the regulation of gonadal development in *P*. *trituberculatus*.

Gene	Sequence ID
3-hydroxy-3-methylglutaryl-coenzyme A synthase	comp125439_c0
3-hydroxy-3-methylglutaryl-coenzyme A reductase	comp120111_c1
Farnesyl pyrophosphate synthase	comp129671_c0
Farnesoic acid O-methyl transferase	comp47818_c0; comp115960_c0
JHE-like carboxylesterase 1	comp103194_c0
JHE-like carboxylesterase 2	comp129436_c0
Methoprene-tolerant	comp129328_c0
Ecdysteroid receptor	comp110985_c0; comp124507_c1; comp124507_c3
Retinoid X receptor	comp123143_c1
E75 nuclear receptor	comp110276_c0
HR3 nuclear receptor	comp117779_c2
3β-hydroxysteroid dehydrogenase	comp121116_c0
17β-hydroxysteroid dehydrogenase type 3	comp117285_c0; comp126737_c1
17β-hydroxysteroid dehydrogenase type 8	comp116427_c0; comp130223_c1; comp105537_c0
Membrane progestin receptor γ	comp115789_c0
Progestin membrane receptor component 1	comp105610_c2
Cyclooxygenase	comp123466_c0; comp127940_c0
Prostaglandin D synthase	comp73990_c0; comp87538_c0
Prostaglandin E synthase	comp123337_c0
Prostaglandin E2 receptor	comp121411_c0
5-hydroxytryptamine receptor	comp116348_c0; comp1021305_c0; comp112658_c1
Dopamine receptor	comp124753_c0
Octopamine receptor	comp118564_c1; comp127071_c0; comp81773_c0;
	comp51865_c0; comp123122_c0

Methyl farnesoate (MF), a crustacean juvenile hormone (JH) analogue, is crucial for the regulation of reproductive processes, such as sex determination, ovarian maturation and testicular development [4,50–53]. Previous studies showed that MF level was correlated with reproductive development [54], and its level was modulated by changes in the rates of both biosynthesis and degradation. The pathway for MF biosynthesis is similar to the mevalonate pathway for acyclic isoprenoids [55,56]. During the initial steps of this pathway, mevalonate is synthesized from acetate by 3-hydroxy-3-methylglutaryl-coenzyme A synthase (HMGS) and 3-hydroxy-3-methylglutaryl-coenzyme A reductase (HMGR), and converted to isopentyl pyrophosphate. Then isopentyl pyrophosphate are condensed by farnesyl pyrophosphate (FPP) synthase to form FPP, and subsequently FPP is hydrolyzed to farnesol and oxidized to form farnesoic acid. Finally, FA is converted to MF *via* farnesoic acid O-methyl transferase (FAOMeT). Four genes encoding the enzymes mentioned above, including HMGS, HMGR, FPP synthase and FAOMeT, were found in this transcriptome. Compared with the synthetic pathway of MF, much less is known about its degradation. The degradation of MF is considered to be similar to that of JH, which requires carboxylesterase-catalyzed ester hydrolysis. Recently, two JH esterase-like carboxylesterases were cloned in the shrimp *Pandalopsis japonica* [57]. Here we found orthologues of these two genes in our transcriptome, and the expression of them were abundant in both ovary and testis. This result indicated that gonad was a major site for MF catabolism in *P*. *trituberculatus*, which was consistent with previous findings in other crustancean species, such as *Libinia emarginata* and *Procambarus clarkii* [58,59]. In addition, an orthologue of Methoprene-tolerant (Met) which functions as a receptor for MF, mediating the physiological effects of MF in crustaceans [60], was also identified in this transcriptome.

Ecdysteroids, synthesized in Y-organ, were primarily considered to be molting hormones, however, recent studies have demonstrated that they also played a major role in regulating vitellogenesis, ovarian maturation and spermatogenesis in decapod crustaceans [42,61,62]. Ecdysteroid signaling is mediated through its nuclear receptors which act as ligand-dependent transcription factors [63]. Ecdysteroids bind to the heterodimer formed by ecdysteroid receptor (EcR) and retinoid X receptor (RXR), which in turn activates transcription of responsive genes and initiates an ecdysteroid cascade reaction [64]. In this transcriptome, the nuclear receptors, EcR and RXR were identified. In addition, homologs of two ecdysteroid-response transcription factors, HR3 and E75, were also found, both of which have been reported to be critical in vitellogenesis and oogenesis in insects [65]. Recent studies showed that HR3 and E75 in macrocrustacean *Daphnia magna* have high similarity in structure and function with their orthologues in insects [66], therefore these two genes may participate in the regulation of gonadal development in crustaceans.

Vertebrate-type steroid hormones, such as 17β-estradiol, progesterone and testosterone, have been reported to be present in crustaceans [13,67,68] and implicated in the regulation of ovarian growth, vitellogenesis and spermatogenesis [69–72]. Previous studies have shown that crustacean gonad tissue is a major site for the biosynthesis of these steroid hormones, in which activities of several key steroidogenic enzymes, such as 3β-hydroxysteroid dehydrogenase (3β-HSD) and 17β-HSDs, were detected. In this gonad transcriptome, we identified genes encoding 3β-HSD and two types of 17β-HSD (type 3 and 8). In vertebrates, 3β-HSD the conversion of Δ^5^–3β-hydroxysteroids into Δ^4^-3-ketosteroids which is necessary for the formation of all classes of steroid hormones [73]. 17β-HSD type 3 is responsible for testosterone biosynthesis, which catalyzes the conversion of androstenedione into testosterone, while 17β-HSD type 8 catalyzes the oxidation of 17β-estradiol, testosterone and dehydroepiandrosterone, and the reduction of estrone to 17β-estradiol [74,75]. Apart from the genes involved in the steroid hormone synthesis, genes encoding their receptors were also found here, including *membrane progestin receptor γ* (*mPRγ*) and *progestin membrane receptor component 1* (*PGMRC1*), both of which play important roles in mediating the rapid nongenomic signaling of progestin in vertebrates [76]. In this study, the expression of these two genes in ovary was significantly higher than that in testis, which indicated their potential implication in ovarian development in *P*. *trituberculatus*.

Prostaglandins (PGs) comprise a family of lipid-derived autacoids, and some of them, namely PGD2, PGE2 and PGF2α, have been proven to be involved in vitellogenesis, oocyte maturation and ovulation in crustacean species [77,78]. In the present study, we identified three genes related to the biosynthesis of these PGs, including *cyclooxygenase* (*COX*), *PGD synthase* (*PGDS*) and *PGE synthase* (*PGES*), and one gene encoding prostaglandin E2 receptor EP4 (*PTER4*) (Table 3). COX catalyzes the conversion of arachidonic acid (AA) into PGH2 which is the rate-limiting step for PGs biosynthesis. PGDS and PGES can convert PGH2 to PGD2 and PGE2, respectively. PGER4 is reported to mediate actions of PGE2 in oocyte maturation in mammals [79]. Previous studies in crustaceans mainly focused on regulatory roles of PGs in ovarian development [80], however, in this study all these PGs-related genes showed testis-biased expression, suggesting the possible involvement of PGs in regulatory events associated with testicular development in *P*. *trituberculatus*.

**Table 3:** Real-time PCR confirmation of DEGs between ovary and testis.

Sequence ID	Gene	Illumina sequencing Testis/Ovary	Real-time PCR Testis/Ovary
comp115789_c0	Membrane progestin receptor γ	0.23	0.20
comp105610_c2	Progestin membrane receptor component 1	0.22	0.26
comp127940_c0	Cyclooxygenase	5.97	6.71
comp73990_c0	Prostaglandin D synthase	2.58	2.99
comp123337_c0	Prostaglandin E synthase	7.22	8.19
comp121411_c0	Prostaglandin E2 receptor	8.31	2.69
comp123711_c0	Follistatin	0.16	0.12
comp128371_c0	Forkhead box L2	0.08	0.10
comp105761_c0	Mothers against decapentaplegic homolog 3	0.22	0.58
comp111219_c0	Feminization-1	8.05	7.18
comp127112_c0	SRY-related HMG-box gene 9	93.63	9.06
comp89760_c0	Wingless-type MMTV integration site family, member 4	0.13	0.11

Biogenic amine neurotransmitters, including 5-hydroxytryptamine (5-HT), dopamine (DA) and octopamine (OA), have been found to serve diverse roles in reproduction of decapod crustaceans [81–83]. 5-HT can induce oocytes and ovarian growth in females and testicular maturation in males [84–86], while DA and OA were reported to delay gonadal development [52,71]. The biogenic amines exert the regulatory effects *via* specific cell-surface receptors, the majority of which belong to the superfamily of G-protein-coupled receptors. [87]. In this study, the receptors for 5-HT, DA and OA were identified, which will enable us to investigate the signal transduction cascades through which the biogenic amines regulate reproductive processes.

In this transcriptome, we identified a number of genes potentially involved in the regulation of gonadal development and maturation in *P*. *trituberculatus*, some of which were discovered in crustaceans for the first time, such as *17β-HSDs* and *mPRγ*. More detailed studies are required to elucidate their roles in gonadal development and maturation in this species.

### Differentially expressed genes between ovary and testis

The identification and characterization of differentially expressed genes (DEGs) between the ovary and testis is of vital importance for the understanding of the regulatory mechanisms controlling differentiation and development of gonads. In the present study, statistical analysis produced 5,919 genes exhibiting differential expression between ovary and testis (*q* value<0.005 & |log2 (fold change)|>1), of which 1,000 were up-regulated in the ovary and 4,919 were down-regulated. Due to the lack of genomic information for crustacean species, a large fraction of DEGs (64.52%) cannot be annotated, which may contain novel genes important for gonadal differentiation and development. Further studies are necessary to functionally characterize these genes. Among those annotated DEGs, many genes related to oogenesis or spermatogenesis were identified (S1 Table), such as genes associated with vitellogenesis (*Vitellogenin*, *Vitellogenin receptor*, *Vigilin* and *Vitelline membrane outer layer 1-like protein*), oocyte maturation (*Cyclin B* and *Cell division cycle protein 2*), spermatocytogenesis and spermatidogenesis (*Dmc 1* and *Synaptonemal complex protein 1* and *2*), ubiquitin proteolytic system (*E3 ubiquitin-protein ligase Ubr2*, *SUMO-1*, *E3 SUMO-protein ligase RanBP2* and *NSE2*, *SUMO-activating enzyme subunit 1* and *subunit 2*, and *Ubiquitin-conjugating enzyme E2*), and so on. In addition to the gametogenesis-related genes, we also found several genes which were previously reported to play key roles in regulating gonadal differentiation and development in invertebrates and nematodes, including *Forkhead protein l2* (*Foxl2*), *Wnt4* and *Follistatin* (*Fst*), *Feminization-1* (*Fem-1*), *Mothers against decapentaplegic homolog 3* (*Smad3*) and *SRY-related HMG-box gene 9* (*Sox9*).

*Foxl2*, encoding a forkhead transcription factors, is one of the most conserved genes controlling the differentiation and development of the ovary in vertebrates [88,89]. In this study, *Foxl2* was predominantly expressed in ovary, indicating its potential implication in ovarian differentiation and development of *P*. *trituberculatus*. As a member of the Wnt family, Wnt4 has been well documented to play a crucial role in female reproductive development in mammals by regulating Müllerian duct formation, controlling steroidogenesis in the gonad and supporting oocyte development [90]. Deficiency of *Wnt4* in mice resulted in a dramatic reduction in the number of developing oocytes, and gives rise to masculinization of the female gonad [91]. Fst, a secreted glycoprotein, is known to be critical in regulating folliculogenesis and the development of ovary by neutralizing the autocrine-paracrine action of Activin in promoting the differentiation and proliferation of granulosa cells [92]. Smad3, an important mediator of the TGF-β signaling pathway, is essential in regulating the response of ovary to follicle-stimulating hormone during folliculogenesis [93,94]. The higher expression of *Wnt4*, *Fst* and *Smad3* in this transcriptome suggested that they may participate in the regulation of ovarian development in the swimming crab.

In nematodes, Fem-1 functions in a signaling pathway that controls sex determination, whose expression is essential for achieving all aspects of the male phenotype [95]. In this study, orthologues of *Fem-1* were found and exhibited testis-biased expression, implying their implication in testicular development and spermatogenesis. Sox9, a HMG-box transcription factor, has been reported to be critical in testis differentiation and development in vertebrates [96]. The higher expression of *Sox9* in testis compared with that in ovary was observed in this study, which indicated that it may be involved in the differentiation and development of testis tissue in this species.

In order to validate expression profiles obtained from Illumina sequencing analysis, twelve DEGs were chosen for qRT-PCR analysis using the same RNA samples. Of these, nine genes closely matched the results detected by Illumina sequencing (Table 3). Although the other three did not perfectly match to the sequencing data, the up- or down-regulated trends were similar. In general, the qRT-PCR results were in good agreement with the Illumina sequencing analysis, which indicated that the Illunima data was credible.

### Putative molecular markers

**Fig 4:**
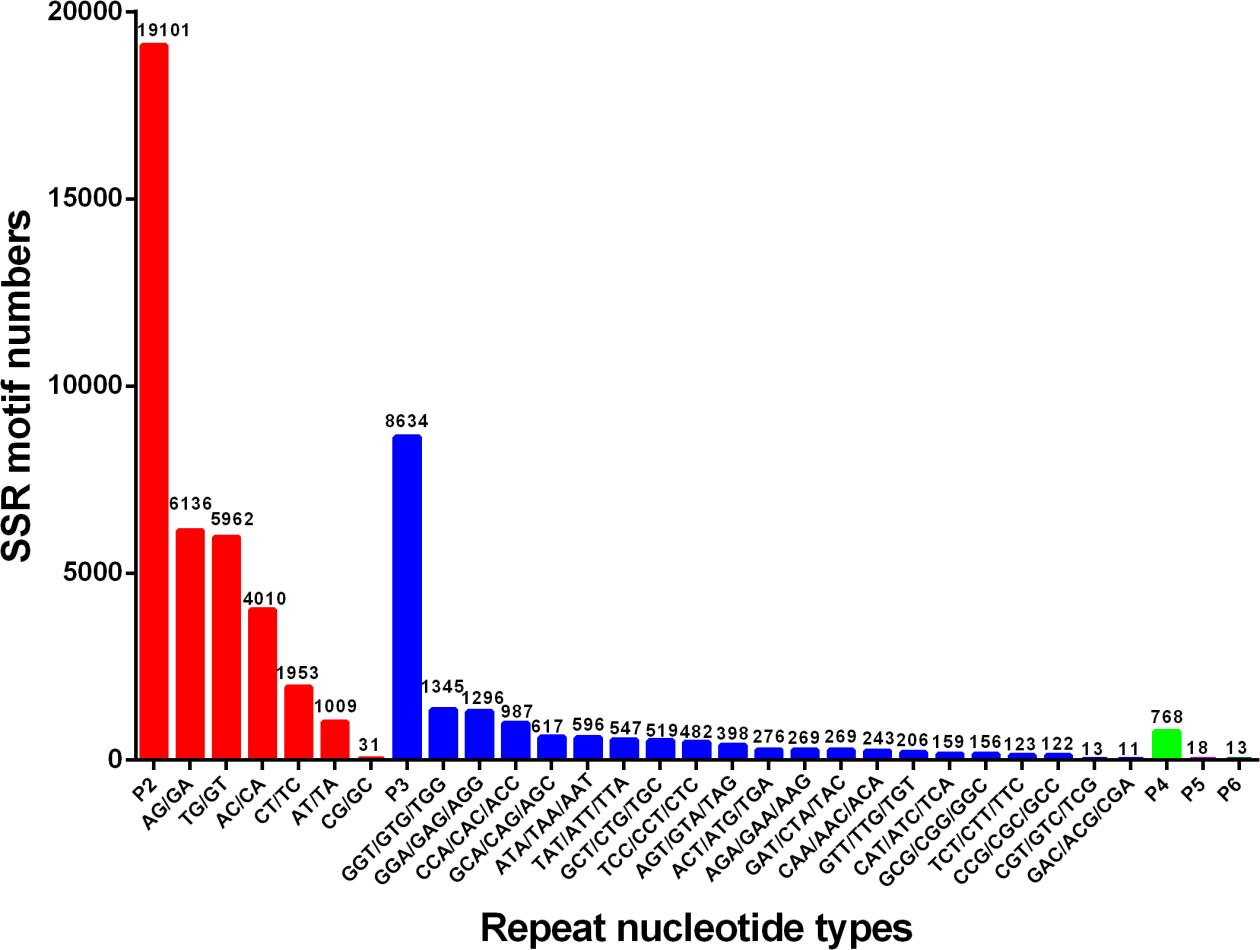
Distribution of identified SSRs according to motif types.

**Fig 5:**
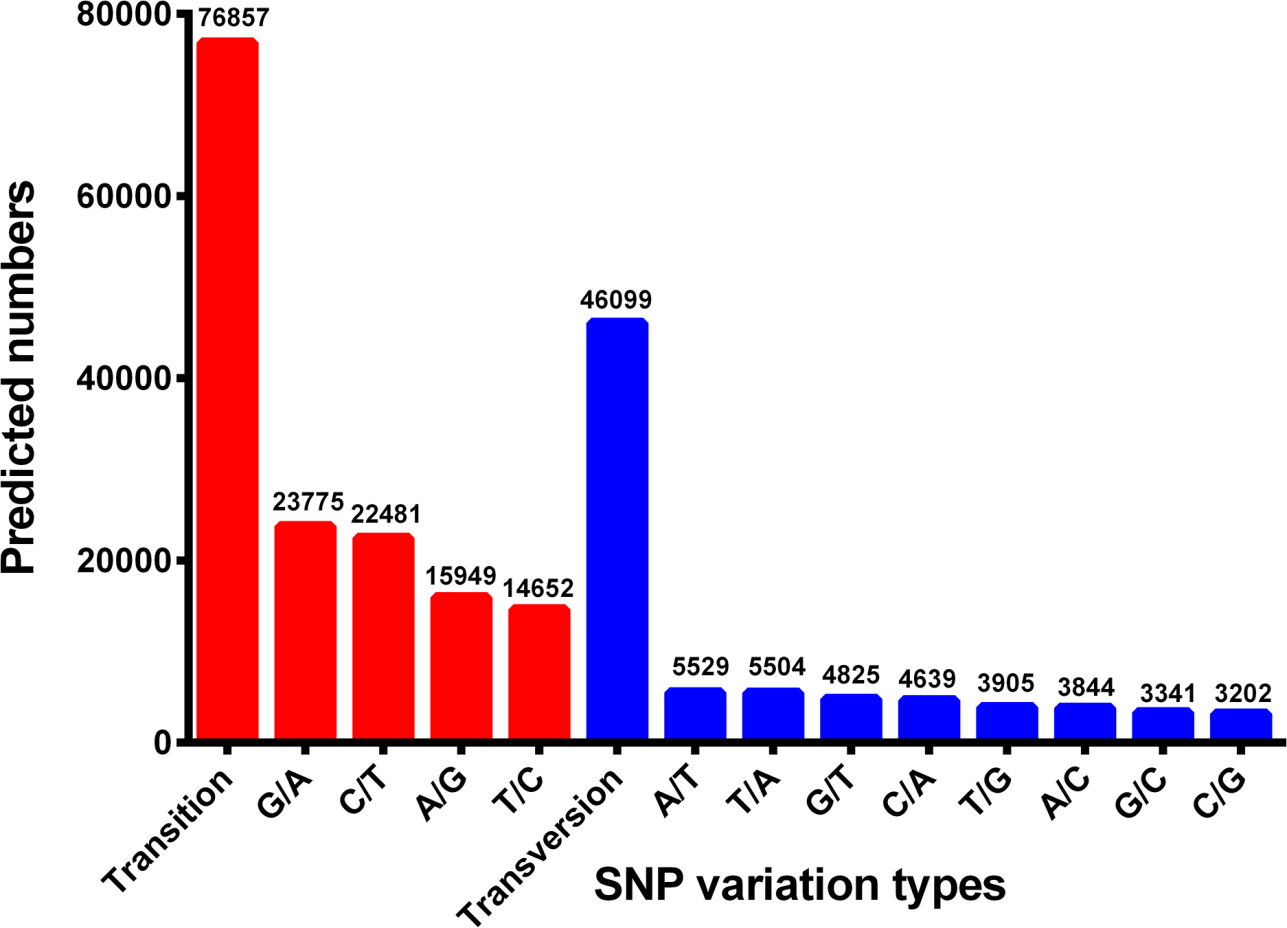
Distribution of putative SNPs in *P*. *trituberculatus* sequences.

Transcriptome sequencing is a rapid and cost-efficient approach for development of genetic markers. Among the various molecular markers, SSRs have a wide range of applications such as parentage analysis, marker assisted selection (MAS), quantitative trait loci (QTL) association and population genetics, by virtue of their highly polymorphic and codominant nature [97,98]. To identify SSRs, all the unigenes in this transcriptome dataset were searched with perl script MISA. A total of 28,534 SSRs were identified in 22,627 unigenes with the frequency of one SSR per 5.09 kb of the unigenes. The density was higher than those previously reported for *Scylla paramamosain* (1/12.08 kb) and *Macrobrachium nipponense* (1/5.70 kb) [19,20]. As shown in Fig 4, of all the SSRs, the most abundant type of repeat motif was di-nucleotide repeats (19,101), accounting for 66.94%, followed by tri- (8,634), tetra- (768), hexa-nucleotide (18) and penta- (13) repeat units. Among the di-nucleotide repeats motifs, (AG/GA)n, (TG/GT)n, and (AC/CA)n were the dominant types with the frequencies of 32.12%, 31.21% and 20.99%, respectively. The most common tri-nucleotide repeats motifs was (GTG/GGT/ TGG)n (15.58%), followed by (GAG/GGA/ AGG)n (15.01%) and (CAC/ ACC / CCA)n (11.43%). These results were different from those reported in *S*. *paramamosain* and *M*. *nipponense* [19,20], indicating that SSR repeat types may be species-specific in decapod crustaceans. In addition to SSRs, by mapping against 54,960 reference unigenes we also obtained a total of 111,646 putative SNPs, wherein 76,857 were transitions (Ts) and 46,099 were transversions (Tv), yielding a Ts: Tv ratio of 1.67: 1 across the *P*. *trituberculatus* gonad transcriptome. The GA/AG, TC/CT and TA/AT SNP types were the most common, while CG/GC types were the least SNP types (Fig 5). In order to assess the reliability of the putative SNPs, thirty-five of these SNPs were selected randomly for validation with PCR amplification and Sanger sequencing, and twenty-five of them (71.42%) were validated (S3 Table). Generally, the successful validation for the majority of putative SNPs confirmed the utility of mining Illumina transcriptome sequence for SNPs. In the present study, a large number of SSRs and SNPs were identified from Illumina sequencing data. It is envisaged that the markers will provide an invaluable resource for population genetics, genetic mapping, QTL association and evolutionary studies in *P*. *trituberculatus*.

## Conclusion

This study represents the first utilization of Illumina sequencing technology to conduct a comprehensive transcriptome analysis of *P*. *trituberculatus* gonad. Our transcriptome sequencing generated a total of 54,960 unigenes, among which many genes potentially involved in gonadal development and maturation were identified. This transcriptome dataset will enrich the genomic information for *P*. *trituberculatus*, and provide a fundamental support for future research on the molecular mechanisms governing gonadal development of this species. In addition, a large number of putative SSRs and SNPs were obtained, which should be useful as molecular markers for functional genomics and breeding research in this species and other closely related species.

## Supporting Information

S1 Fig Sequence length distribution of transcripts assembled from Illumina reads. (TIFF)

S1 Table DEGs related to oogenesis and spermatogenesis. (XLSX)

S2 Table Primers used in Real-time PCR confirmation of DEGs. (XLSX)

S3 Table Validation of putative SNPs in the transcripts of *P*. *trituberculatus*. (XLSX)
