# Correction: *De novo* Transcriptome Analysis of *Portunus trituberculatus* Ovary and Testis by RNA-Seq: Identification of Genes Involved in Gonadal Development

**DOI:** 10.1371/journal.pone.0133659

**Published:** 2015-07-15

**Authors:** The PLOS ONE Staff

The first author, Xian-liang Meng, is incorrectly noted as a corresponding author of this article. The publisher apologizes for the error.
