# Supplementary figures and images for: De novo Transcriptome Analysis of Portunus trituberculatus Ovary and Testis by RNA-Seq: Identification of Genes Involved in Gonadal Development
Source: PLoS One. 2015 Jun 4;10(6):e0128659. doi: 10.1371/journal.pone.0128659 (PMC4456094; doi:10.1371/journal.pone.0128659)

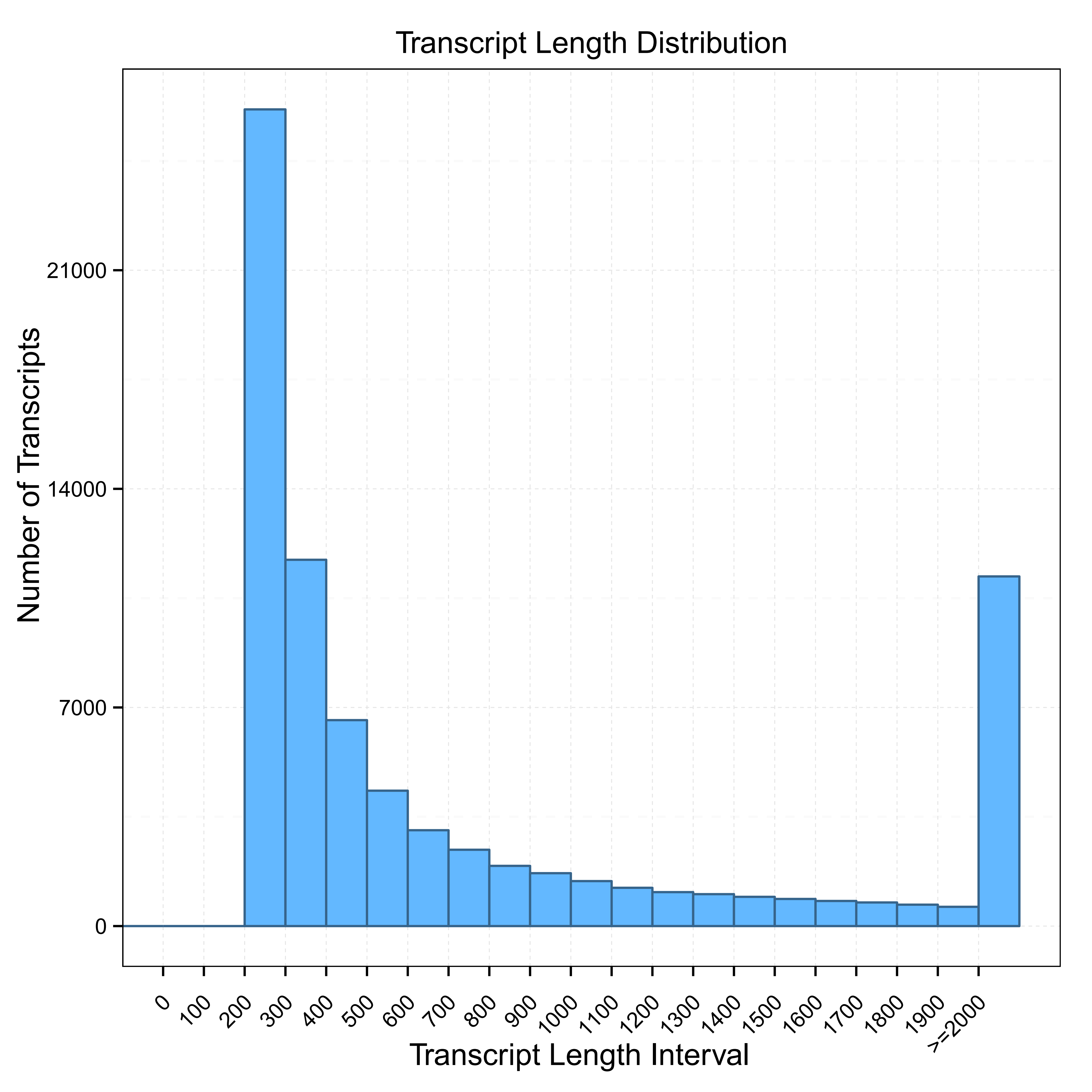

Supplement: S1 Fig — (TIFF) [file pone.0128659.s001.tiff]
